# Risk stratification-based tiered medication therapy management for outpatients with asthma: a practical prospective study

**DOI:** 10.3389/fphar.2026.1848325

**Published:** 2026-07-15

**Authors:** Yufei Lian, Shujuan Xiong, Haijing Zhao, Yanru Deng, Huizhen Wu

**Affiliations:** 1 Key Laboratory of Clinical Pharmacy of Hebei Province, Department of Pharmacy, Hebei Provincial People’s Hospital, Shijiazhuang, Hebei, China; 2 Department of Pharmacy, Zhangjiakou Municipal Hospital of Traditional Chinese Medicine, Zhangjiakou, Hebei, China; 3 Office of Scientific Research, Hebei Provincial People’s Hospital, Shijiazhuang, Hebei, China

**Keywords:** asthma, cough and asthma pharmacy clinic, pharmaceutical care, risk stratification, tiered medication therapy management

## Abstract

**Objective:**

This study aims to explore a new model of tiered medication therapy management (MTM) services for outpatients with bronchial asthma and evaluate its effectiveness.

**Methods:**

A new model of tiered MTM services for outpatients with asthma (referred to as the T-MTM model) was established based on the triangle risk stratification model, and its multidimensional value was evaluated using the ECHO model. A single-center, prospective, randomized controlled, open-label study was conducted. A total of 126 asthma patients who visited the Medication Therapy Clinic at Hebei Provincial People’s Hospital from January 2024 to December 2024 were selected and randomly divided into an observation group and a control group, with 63 patients in each group. Patients in the observation group received T-MTM services, while those in the control group received traditional MTM services. The differences in values across the three dimensions—economic, clinical, and humanistic—were compared between the two groups before and after the intervention.

**Results:**

Before the intervention, there were no statistically significant differences in any indicators between the two groups (*p* > 0.05). After the intervention, the patients in the observation group who received T-MTM services demonstrated superior outcomes in economic, clinical, and humanistic values than in those in the control group, with statistically significant differences (*p* < 0.05). The mean difference in the cost-utility ratio is as follows: MD = −7.54 (95% CI, −9.38 to −5.70), whereas that in the ACT score is given as MD = 2.25 (95% CI, 1.41–3.09). The improvement in the observation group was 5.05 points, exceeding the minimum clinically important difference (MCID) threshold of 3 points (MARS-A adherence score: MD = 0.44 [95% CI, 0.25–0.63]). The average duration of a single service per pharmacist was reduced by 18.92 min, representing an efficiency improvement of approximately 35%.

**Conclusion:**

Risk-stratified tiered MTM services enable pharmacists to rapidly identify high-risk asthma patients, significantly improve work efficiency and service volume, and simultaneously enhance patients’ self-management capabilities. This approach provides evidence-based guidance for optimizing and tiering outpatient asthma pharmacy service resources and represents a modified pharmacy service model worthy of promotion.

## 1 Introduction

Bronchial asthma (BA) is a global chronic airway disease. As of 2025, there are approximately 300 million people with asthma worldwide, of whom only approximately 50%–60% have their condition effectively controlled (Papi et al., 2018). Statistics indicate that the primary causes of poor asthma control include self-discontinuation of treatment, incorrect inhalation techniques, and intolerance to medication-related adverse reactions (Malaya et al., 2024). Medication therapy management (MTM) refers to services provided by professionally trained MTM pharmacists, including recommendations for treatment plan adjustments, medication education, medication counseling, and guidance on self-medication (Pellegrino et al., 2009). Due to its professional, detailed, and personalized dynamic management approach, it is widely used in the management of patients with chronic conditions such as asthma (Liu et al., 2022; George, 2018). However, this traditional MTM service, originating in the United States, has increasingly revealed challenges in the practice of pharmaceutical care for patients with chronic diseases in China (Mehuys et al., 2008). Due to the limited number of pharmacists specializing in cough and asthma management in China, the service model involves multiple steps and is time-consuming, resulting in a limited number of patients being served by pharmacists; consequently, many patients do not have the opportunity to access equitable pharmaceutical care resources. Furthermore, the cumbersome nature of the service model and the time-consuming and labor-intensive documentation process lead to an excessive workload for pharmacists. Consequently, pharmacists both domestically and internationally have begun exploring ways to optimize the MTM service model. The triangle management model, which scientifically stratifies and categorizes patients based on disease risk factors, has been widely applied in studies aimed at improving MTM services for chronic diseases such as hypertension and diabetes due to its ability to significantly enhance service efficiency and standardization. For example, Ye et al. (2024) applied the triangle theory to hypertension management and achieved preliminary results. Chen et al. (2019) implemented tiered management of diabetic foot patients based on the triangle stratification, achieving significant results in improving disease control rates, enhancing adherence, and reducing the duration of pharmacist services. However, research on modified MTM service models for asthma patients remains limited.

Starting in October 2022, pharmacists at our hospital began exploring a new tiered MTM service model based on the triangle risk stratification model (referred to as the T-MTM service model) among outpatient asthma patients. Through stratified patient management and tiered service delivery, pharmacists have streamlined unnecessary service steps and reduced the time spent on each patient, thereby improving both asthma control rates and patients’ self-management capabilities. This model has been implemented in numerous hospitals across China, demonstrating significant results across multiple evaluation metrics, including economic, clinical, and patient-centered outcomes. In summary, the new T-MTM service model overcomes the shortcomings of traditional MTM services and fills the gap in China for a novel, risk-stratified, improved medication therapy management service model for outpatient asthma patients.

This study, based on a single-center randomized controlled design and utilizing the ECHO multidimensional evaluation system, systematically compares the differences between T-MTM tiered services and traditional MTM. Its objectives are (1) to establish a standardized model for the “triangle” risk stratification MTM tiered service for outpatient asthma; (2) to validate the practical value of the new model from economic, clinical, and patient-centered perspectives; and (3) to provide evidence-based support for the stratified management of outpatient asthma pharmaceutical services and the optimal allocation of healthcare resources.

## 2 Materials and methods

### 2.1 Study design

This study is a single-center, prospective, randomized controlled, open-label trial. The study protocol was approved by the Institutional Review Board of our hospital [Ethics Approval: (2023) Research Ethics Review No (112)], and all patients signed informed consent forms.

### 2.2 Study population

The study included 126 adult patients with bronchial asthma who visited our hospital and received services from the Cough and Asthma Pharmacy Clinic between January 2024 and December 2024.

Inclusion criteria are as follows: (1) age 18–70 years, (2) adult patients diagnosed with asthma by a physician according to the 2025 Global Initiative for Asthma (GINA) guidelines, and (3) patients who voluntarily agreed to participate in the study and were able to cooperate throughout the entire cough and asthma pharmaceutical care service. Exclusion criteria are as follows: (1) patients with concurrent chronic airway diseases, such as chronic obstructive pulmonary disease (COPD), pulmonary interstitial fibrosis, or chronic bronchitis; (2) patients with other underlying conditions that prevent them from completing relevant examinations, assessment scales, or follow-up visits; (3) pregnant or lactating women; (4) patients with cognitive impairment or psychiatric disorders; and (5) patients without contact information. Withdrawal criteria are as follows: (1) participants who are unable to complete follow-up for any reason, (2) participants who experience a death endpoint prior to the end of the trial, and (3) participants who request to withdraw from the trial prior to its conclusion.

### 2.3 Sample size calculation

According to the experimental design, the sample size formula is n_1_ *=* n_2_ = [Z_a/2_ $\sqrt{2p\left(1‐p\right)}$ +Z_β_ $\sqrt{p1\left(1‐p1\right)+p2\left(1‐p2\right)}$ ]^2^/**(**p_1_ − p_2_ **)** ^2^, where *n* is the sample size; a is the significance level; when a = 0.05, Z_a/2_ = 1.96; 1 − β is the power of the test; when β = 0.1, *Z*_β_ = 1.28; p is the sample proportion, defined as p = (p_1_ + p_2_)/2. According to the findings of the 2019 study “Prevalence, Risk Factors, and Current Status of Asthma Management Among Chinese Adults” (Ye et al., 2024), the asthma control rate among Chinese adults is 28.6%, so p_2_ = 0.30, Based on previous studies (Chen et al., 2019; Global Initiative for Asthma, 2025), MTM services are estimated to improve adult asthma control rates by approximately 60%, so p_1_ = 0.60. Applying the formula and rounding to the nearest whole number yielded a minimum sample size of 56 participants per group (n_1_ = n_2_ = 56). Estimating a 10% dropout rate requires each group to have a minimum sample size of n/0.9 = 62.22, which was rounded to 63 participants.

### 2.4 Randomization and blinding

Participants were randomized using a random number table (1:1) into an observation group and a control group, with 63 participants in each group. The random sequence was generated by an independent statistician, and patients were numbered in the order of their visits. Given the nature of pharmaceutical services, it was not possible to implement blinding for patients and pharmacists (open-label design). However, data analysts remained blinded to the group assignments to minimize detection bias. Additionally, standardized protocols were strictly followed during the administration of subjective questionnaires to ensure consistency.

### 2.5 Intervention measures

The control group received monthly in-person MTM services provided by pharmacists (with additional visits available at any time during acute exacerbations), while the observation group received tiered MTM services based on the triangle risk stratification model (referred to as T-MTM services), tailored by pharmacists according to the patients’ conditions.

The fee structure for both groups was 10 yuan per person per visit for in-person consultation registration fees, with no restrictions on service duration or scope; telephone follow-ups were provided at no cost per person per visit.

### 2.6 Establishment and implementation of the T-MTM service model

#### 2.6.1 Identification of risk factors and weighting

The Delphi method was used to conduct expert consultations and consistency testing. The factors were formally confirmed following a written survey and voting process by the expert panel of our hospital’s Pharmaceutical Affairs Management Committee. The specific process is as follows: A literature review conducted to search domestic and international guidelines, expert consensus documents, and systematic reviews on asthma aimed to identify risk factors for poor asthma control. The included literature was synthesized and analyzed to identify the top five most frequently cited factors: disease severity (ACT score), number of comorbidities, liver and kidney function, number of medications used, and inhaler adherence. These five factors were assigned equal weights of 1:1:1:1:1, as determined following three rounds of expert discussions. The grading criteria for these five risk factors were established based on recommended values from international standards for disease diagnosis and treatment or authoritative clinical guidelines (e.g., an ACT score ≤19 was defined as uncontrolled).

#### 2.6.2 Establishment of T-MTM service stratification and grading criteria

**FIGURE 1:**
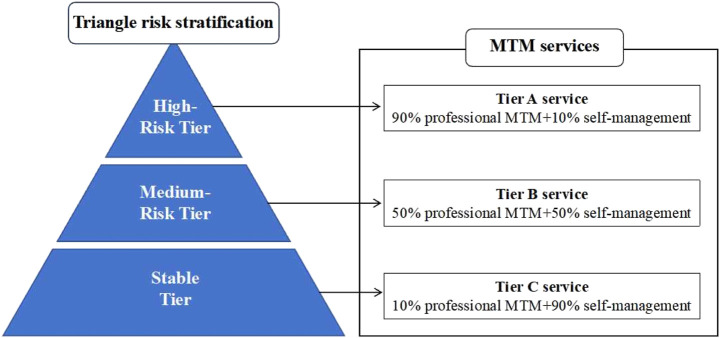
Triangle-based tiered MTM service model.

Based on the triangle chronic disease risk stratification model proposed by Kaiser Permanente (de Dios Lopez et al., 2025), this study conducted a systematic literature review and three rounds of expert Delphi surveys to ultimately identify the following core variables: Combining five factor-disease severity, the number of comorbidities, pathophysiological status, the number of medications used, and inhaler adherence, asthma patients are classified into three tiers: severe, high-risk, and stable (Table 1). Professional pharmaceutical care services are then allocated proportionally based on the treatment needs of patients in each tier: (1) the severe group receives Level A pharmaceutical services, consisting of 90% professional medication therapy management and 10% self-management; (2) the high-risk group receives Level B pharmaceutical services, consisting of 50% professional medication therapy management and 50% self-management; and (3) the stable group receives Level C pharmaceutical services, consisting of 10% professional medication therapy management and 90% self-management, forming a pyramid-shaped MTM tiered service model (Figure 1).

**TABLE 1:** Triangle-based tiered MTM service standards for outpatient asthma patients.

Triangle stratification	Asthma severity		Number of comorbidities	Number of medications used	Pathophysiological status		TAI score	MTM Tier
	Asthma control	ACT score			Liver function (Child–Pugh score)	Renal function (creatinine clearance)		
High-risk tier	Uncontrolled	5–15 points	>5 types	≥10	≥10 points	≤30 mL/min	<45 points	Tier A pharmaceutical service
Medium-risk tier	Partially controlled	16–20 points	3–5 types	5–9	7–9 points	31–89 mL/min	46–49 points	Tier B pharmaceutical service
Stable tier	Fully controlled	21–25 points	<3 types	<5	5–6 points	≥90 mL/min	50 points	Tier C pharmaceutical service

1 is the primary criterion; 2–5 are secondary criteria. A patient is classified within this tier if they meet criterion 1 or three of the criteria listed in 2–5. ACT, asthma control test; TAI, inhaler adherence test.

#### 2.6.3 Implementation of T-MTM service tiering and categorization criteria

The stratification of all patients is independently performed by two trained clinical pharmacists. In the event of a disagreement, a third senior pharmacist is consulted, and the decision is made by majority vote. Following stratification, all patients receive MTM services corresponding to their final tier; no patient is excluded or reclassified on the grounds of “not fully meeting all criteria.” Additionally, to avoid bias, pharmacists do not discuss the details of group interventions with one another during the service period.

#### 2.6.4 Feasibility validation of the T-MTM service model

Prior to the formal trial, this study conducted a single-center prospective pilot study to validate the model’s reliability and validity. Fifty asthma patients were selected for a 6-month pilot phase. The results demonstrated that the stratified system exhibited good internal consistency and clinical feasibility. Based on feedback from the pilot study, certain intervention processes were optimized and subsequently implemented in the formal study.

#### 2.6.5 Development and implementation of tiered MTM service content

Pharmacists provide standardized MTM services at different levels based on patient risk stratification. The primary differences between service tiers lie in the frequency of MTM services and the degree of patient self-management. High-risk patients, due to poor overall disease control and a higher likelihood of acute exacerbations, receive services primarily involving pharmacist intervention supplemented by patient self-management, with MTM services provided once a month. Patients in the moderate-risk tier have a lower risk of disease progression and acute exacerbations compared to those in the high-risk tier. Pharmacist interventions are reduced, while patient self-management is increased; MTM services are provided once every 2 months. Patients in the stable-risk tier have relatively stable conditions and a low risk of acute exacerbations. These services are primarily based on patient self-management, with pharmacist interventions serving as a supplement; MTM services are provided once every 3 months (Table 2). Patients’ MTM service levels are dynamically adjusted. After each service, pharmacists assess the patient’s tier using the triangle classification criteria and schedule the next MTM service at the appropriate level based on the new tier, thereby achieving dynamic management.

**TABLE 2:** Triangle-based tiered MTM service content.

Service content		Tier A		Tier B		Tier C	
		Service method	Service frequency	Service method	Service frequency	Service method	Service frequency
Pharmacological consultation	1. Consultation on asthma control	In-person consultation/home visit	Initial visit During acute exacerbations Routine: once a month	In-person consultation/home visit	Initial visit During acute exacerbations Routine: once every 2 months	Phone/in-person/home visit	Initial visit During acute exacerbations Routine: once every 3 months
	2. Control of other underlying conditions						
	3. Common ADRs and tolerability of current medications						
	4. Improvement in issues related to previous medication						
Pharmacological assessment	1. ACT score						
	2. Quantitative score for correct use of inhalers						
	3. Assessment of adverse drug interactions						
	4. ADR differentiation						
	5. TAI score						
Medication guidance	1. Recommendations for treatment plan adjustments and communication with physicians						
	2. Education on the proper use of inhalation devices						
	3. Management of adverse drug reactions						
	4. Health education, disease education, and public health outreach						
Patient self-management	Asthma diary (peak flow monitoring/regular medication use, etc.)	In-person consultation/home visit	Patient records, once daily Pharmacist supervision, once a week	Phone/in-person/home visit	Patient records, once daily Pharmacist supervision, once a month	Phone	Patient records, once daily Pharmacist supervision, three times per month

ADR, adverse drug reaction.

### 2.7 Evaluation indicators: the economic-clinical-humanistic model is applied to evaluate the effectiveness of T-MTM tiered services

The primary endpoint of this study was the cost-utility ratio (CUR) in the economic domain; the secondary endpoints included “average service time per pharmacist” in the economic domain, the “ACT score” and “correct inhaler use score” in the clinical domain, and the “MARS-A score” and “quantified asthma health knowledge score” in the humanistic domain.

Multiplicity handling: This study employed a stratified analysis strategy, with the primary endpoint serving as the sole confirmatory hypothesis test target; secondary endpoints were primarily used as supporting evidence, and no statistical adjustments were made for multiple comparisons (to avoid an increased risk of Type II errors due to conservative adjustments).

#### 2.7.1 Economic value evaluation indicators

1. Use a cost-utility ratio model to compare the economic efficiency of the two treatment groups. Cost-utility ratio = patient treatment cost (RMB)/patient health utility value; treatment cost = average daily cost of all medications currently used by the patient (including prescription drugs, over-the-counter drugs, traditional Chinese patent medicines, excluding Chinese herbal decoctions and health supplements that are difficult to calculate) + average daily cost of MTM services. Utility values are derived from the European Quality of Life Questionnaire-5D-5L (EQ-5D-5L) to collect five-digit scores representing patients’ health status across five dimensions: mobility, self-care, usual activities, pain or discomfort, and anxiety or depression. These scores are converted using the Chinese EQ-5D-5L utility value conversion table (Morisky et al., 2008) to calculate the final utility value for each patient; specific conversion methods refer to prior studies (Lian et al., 2024). 2. Compare the time value between the two groups using the average service duration per pharmacist. Average service duration per pharmacist = the average duration of each medication therapy management service session per patient during the follow-up period.

#### 2.7.2 Clinical value evaluation indicators

1. Asthma Control Test (ACT) score: In accordance with the 2025 Global Initiative for Asthma (Global Initiative for Asthma, 2025), the adult ACT questionnaire was used to assess patients’ asthma control before and after the pharmacist intervention. The ACT score ranges from 5 to 25 points, with the following criteria: 25 points indicate well-controlled asthma; 20–24 points indicate partially controlled asthma; and <20 points indicate uncontrolled asthma. The minimum clinically important difference (MCID = 3 points) was determined based on the criteria established by Schatz et al. (2009). 2. Correct use of inhalation devices: We used our hospital’s in-house Correct Use of Inhalation Devices Assessment Scale (Lian et al., 2023), which assigns points to each step of the inhaler’s correct use. The total score is 100 points. Pharmacists score each step based on the patient’s demonstration of device use, and the final score for correct inhaler use is the sum of the scores for each step. Evaluation criteria: 100–90 points, excellent; 89–70 points, good; 69–60 points, pass; and <60 points, fail.

#### 2.7.3 Humanistic value assessment indicators

1. Medication Adherence Report Scale for Asthma (MARS-A): The MARS-A is a specialized scale for asthma patients using inhaled medications, designed to better reflect adherence to inhaled therapy. The scale consists of 10 questions (Tian and Yu, 2014), each scored on a 1–5 scale; higher scores indicate better adherence, and the final score is the average of the 10 questions. Evaluation criteria: ≥4.5 points indicate good adherence; <4.5 points indicate poor adherence. 2. Quantitative assessment of asthma health knowledge: Based on the Delphi method and literature analysis (Ou et al., 2020; Zhu et al., 2016), a self-developed questionnaire on asthma health knowledge was used to assess understanding across five domains: asthma disease awareness, risk factors, symptom management, medication use, and lifestyle. Survey results were quantified on a scale of 20–100 points. Evaluation criteria: 20–59 points indicate low knowledge level (below standard); 60–79 points indicate moderate knowledge level (passing); and 80–100 points indicate high knowledge level (excellent) (Table 3).

**TABLE 3:** Quantitative scoring scale for asthma health knowledge.

No.	Survey question		Quantitative judgment criteria			Score
			1	3	5	
1	Understanding of the disease	Do you think asthma is just a cough?	Yes	Maybe	No	​
		Do you think asthma is bronchitis?	Yes	Maybe	No	​
		Do you think asthma is a psychological disorder?	Yes	Maybe	No	​
		Do you think asthma is a chronic airway disease?	No	Possibly	Yes	​
2	Risk factors	Do you think a cold might make your asthma worse?	No	Possibly	Yes	​
		Do you think smoking, pollen, dust mites, pet dander, cold air, or smog might worsen your asthma?	No	Possibly	Yes	​
		Do you think emotional stress or strenuous exercise might worsen your asthma?	No	Possibly	Yes	​
		Do you think certain medications might make your asthma worse?	No	Possibly	Yes	​
3	Symptom management	Do you know what triggers your asthma attacks?	No	Some	I know them all	​
		Do you know what the common symptoms of an asthma attack are?	0	1 item	≥2 items	​
		Do you know how to monitor your peak expiratory flow rate?	No	Somewhat	I know everything	​
		Do you know the three categories of asthma control?	No	I know a little	I know exactly	​
4	Medication use	Do you think medication is only needed during an asthma attack?	No	Possibly	Yes	​
		Do you think you still need to take medication regularly during remission?	No	Maybe	Yes	​
		Do you believe that inhaled corticosteroids are essential medications during the maintenance phase?	No	Possibly	Yes	​
		Do you believe that asthma can be cured through medication and management?	No	Maybe	Yes	​
5	Self-management	Do you think it is necessary to wear a mask during pollen season, periods of smog, or when exposed to irritating odors?	No	Maybe	Yes	​
		Do you think salbutamol can be used before exercise to prevent asthma attacks?	No	Maybe	Yes	​
		Do you think monitoring peak flow can predict an acute asthma attack?	No	Maybe	Yes	​
		How many treatment goals do you think asthma has? Improving lung function, controlling symptoms, preventing acute exacerbations, reducing complications, and improving psychological status	0–1 items	2–3 items	4–5 items	​
Total						

The asthma knowledge questionnaire is a localized adaptation of an international standard questionnaire, and its reliability and validity have been validated through pilot studies (reliability and validity: Cronbach’s α = 0.85; content validity index (S-CVI) = 0.92). All assessment tools have undergone translation, back-translation, and cultural adaptation to ensure their suitability for use with the Chinese population.

### 2.8 Statistical methods

Statistical analysis of the data was performed using SPSS 28.0 software. The primary analysis was based on the intention-to-treat (ITT) principle. Continuous variables that followed a normal distribution were expressed as x ± s, and comparisons between two samples were performed using t-tests. Continuous variables that did not follow a normal distribution were expressed as M (P25 and P75), and comparisons between groups were performed using the independent samples Mann–Whitney U test. Categorical data are presented as counts or percentages, and comparisons between groups were performed using the chi-square (χ^2^) test. In accordance with the CONSORT 2025 statement (Hopewell et al., 2025), effect sizes and their 95% confidence intervals (CIs) were reported for all key outcome measures. Missing data were primarily handled using a complete-cases analysis (loss to follow-up rate: 2.38%, <5%), supplemented by multiple imputation (m = 20) for sensitivity analysis. The results indicated that the main conclusions remained consistent, indicating the robustness of the study findings.

## 3 Results

### 3.1 Comparison of baseline characteristics

At the end of the study, two patients in the observation group and one patient in the control group were lost to follow-up due to changes in contact information or address, which met the criteria for exclusion. Ultimately, 61 patients were included in the observation group and 62 patients in the control group. All enrolled patients cooperated fully in completing the study, with no recorded deaths and comprehensive data collection. The comparisons of baseline characteristics, including age, gender, disease status, and educational level, between the two groups revealed no statistically significant differences (*p* > 0.05), indicating good comparability between groups. Effect size analysis showed that the 95% confidence intervals for the rate differences or mean differences of all baseline variables included 0, further confirming the balance between the two groups. The results are presented in Table 4.

**TABLE 4:** Comparison of baseline characteristics between the two groups of patients.

Baseline variable	Observation group (n61)	Control group (n62)	Effect size (95% CI)	*p*-value
Gender (male/female)/n	29/32	31/31	−0.032 (−0.187, 0.123)a	0.785
Age (xˉ ± s)/years	64.72 ± 7.89	65.13 ± 8.01	−0.41 (−2.78, 1.96)	0.823
Body mass index (x ± s)/(kg/m^2^)	24.11 ± 2.84	23.76 ± 2.91	0.35 (−0.61, 1.31)	0.531
Educational attainment/[n (%)]				
Junior high school or below	25 (40.98)	24 (38.71)	+2.27% (−11.2, 15.7)	0.797
High school or vocational school	23 (37.71)	25 (40.32)	−2.61% (−14.4, 9.2)	0.766
College	9 (14.75)	11 (17.74)	−2.99% (−12.3, 6.3)	0.653
Graduate and above	4 (6.56)	2 (3.23)	+3.33% (−2.5, 9.2)	0.661
Asthma status	​	​	​	​
Uncontrolled (ACT score: 5–15)	7 (11.48)	8 (12.90)	−1.42% (−10.3, 7.5)	0.809
Partially controlled (ACT score: 16–20)	41 (67.21)	39 (62.91)	+4.30% (−7.2, 15.8)	0.616
Well-controlled (ACT score: 21–25)	13 (21.31)	15 (24.19)	−2.88% (−12.1, 6.3)	0.703
Comorbidities/[n (%)]				
>5 types	12 (19.67)	11 (17.74)	1.93% (−12.34%, 16.20%)	0.780
3–5 types	36 (59.02)	37 (59.68)	−0.66% (−18.23%, 16.91%)	0.941
<3 types	13 (21.31)	14 (22.58)	−1.27% (−17.15%, 14.61%)	0.862
Coronary heart disease	29 (47.54)	27 (43.55)	+3.99% (−10.2, 18.2)	0.657
Diabetes	27 (44.26)	28 (45.16)	−0.90% (−15.1, 13.3)	0.920
Hypertension	44 (72.13)	46 (74.19)	−2.06% (−16.8, 12.7)	0.796
Malignant tumors	16 (26.23)	15 (24.19)	+2.04% (−10.5, 14.6)	0.795
Abnormal liver and kidney function	10 (16.39)	11 (17.74)	−1.35% (−11.2, 8.5)	0.842

### 3.2 Evaluation of ECHO results

#### 3.2.1 Economic value indicators

As shown in Table 5, prior to the intervention, there were no statistically significant differences between the two groups regarding average daily treatment costs, average daily MTM service costs, or cost-utility ratios (*p* > 0.05). After 12 months of the intervention, the average daily treatment cost, average daily cost of MTM services, and cost-utility ratio in the observation group were all significantly lower than those in the control group (MD = −7.54, *p* < 0.001); the average service time per pharmacist was significantly reduced by 18.92 min, and service efficiency improved by approximately 35%. Furthermore, the decline in various patient outcomes was greater after 12 months of the intervention, and the reduction in the observation group was greater than that in the control group. This indicates that as the duration of the pharmacist intervention increases, patient treatment costs decrease further, and the economic burden of disease treatment is reduced.

**TABLE 5:** Comparison of various indicators between the two groups before and after the intervention.

Indicator	Time point	Intervention group (n61)	Control group (n62)	Mean difference/rate difference (95% CI)	Cohen’s d/RR (95% CI)	*p*-value
Economic value						
Average daily treatment cost (RMB)	Before intervention	10.235 ± 3.146	10.127 ± 3.165	0.108 (−0.998, 1.214)	0.034 (−0.272, 0.340)	0.850
	12 months after intervention	6.561 ± 1.936	8.895 ± 2.336	−2.334 (−3.102, −1.566)	−1.085 (−1.412, −0.758)	<0.001
Average daily cost of MTM services (RMB)	Before intervention	0.000 ± 0.000	0.000 ± 0.000	0.000 (0.000, 0.000)	—	1.000
	12 months after intervention	0.137 ± 0.044	0.251 ± 0.096	−0.114 (−0.141, −0.087)	−1.520 (−1.867, −1.173)	<0.001
Cost-utility ratio	Before intervention	41.514 ± 23.446	42.033 ± 26.178	−0.519 (−9.338, 8.300)	−0.021 (−0.327, 0.285)	0.908
	12 months after intervention	10.296 ± 3.838	17.834 ± 6.211	−7.538 (−9.378, −5.698)	−1.476 (−1.819, −1.133)	<0.001
Average duration of each service provided by a pharmacist	Before intervention	54.230 ± 5.786	53.935 ± 5.510	0.295 (−1.718, 2.308)	0.052 (−0.254, 0.358)	0.773
	12 months after intervention	49.787 ± 9.363	30.870 ± 8.461	18.917 (11.187, 26.647)	2.132 (1.738, 2.526)	<0.001
Clinical significance						
ACT score	Before intervention	18.164 ± 4.001	18.097 ± 4.011	0.067 (−1.354, 1.488)	0.017 (−0.289, 0.323)	0.926
	12 months after intervention	23.213 ± 1.684	20.968 ± 2.898	2.245 (1.406, 3.084)	0.943 (0.632, 1.254)	<0.001
	Rangeability (ΔACT)	5.049 ± 3.334	2.885 ± 2.052	2.164 (1.192, 3.136)	0.789 (0.483, 1.095)	<0.001
Score for correct use of the inhaler	Before intervention	71.967 ± 4.655	72.581 ± 5.670	−0.614 (−2.457, 1.229)	−0.119 (−0.424, 0.186)	0.514
	12 months after intervention	95.754 ± 4.048	90.968 ± 5.420	4.786 (3.085, 6.487)	0.997 (0.685, 1.309)	<0.001
	Rangeability (Δscore)	23.787 ± 5.419	18.387 ± 6.289	5.400 (3.308, 7.492)	0.908 (0.600, 1.216)	<0.001
Humanistic value						
MARS-A score	Before intervention	2.902 ± 0.765	2.800 ± 0.722	0.102 (−0.195, 0.399)	0.138 (−0.168, 0.444)	0.801
	12 months after intervention	4.590 ± 0.363	4.151 ± 0.651	0.439 (0.246, 0.632)	0.827 (0.515, 1.139)	<0.001
	≥Proportion of 45 points	41/61 (67.2%)	27/62 (43.5%)	+23.7% (9.6%, 37.8%)	RR = 1.55 (1.16, 2.06)	0.008
Asthma health knowledge Quantitative cognitive level score	Before intervention	59.639 ± 12.945	60.081 ± 12.882	−0.442 (−4.999, 4.115)	−0.034 (−0.340, 0.272)	0.850
	12 months after intervention	83.475 ± 8.656	72.565 ± 12.468	10.910 (7.053, 14.767)	1.012 (0.700, 1.324)	<0.001
	≥Proportion of 80 points	41/61 (67.2%)	29/62 (46.8%)	+20.4% (6.3%, 34.5%)	RR = 1.43 (1.06, 1.92)	0.022

ACT, asthma control test; CI, confidence interval; MARS-A, Medication Adherence Scale for Bronchial Asthma; MD, mean difference; RD, rate difference; RR, relative risk. Cohen’s d: 0.2 indicates a small effect, 0.5 indicates a moderate effect, and 0.8 indicates a large effect.

#### 3.2.2 Clinical value indicators

As shown in Table 5, there were no statistically significant differences in ACT scores or scores for the correct use of inhalers between the two groups prior to the intervention (*p* > 0.05). Twelve months after the intervention, ACT scores in both groups increased compared to preintervention levels; however, the increase in the observation group was significantly greater than that in the control group. Postintervention, ACT scores in the observation group were significantly higher than those in the control group (MD = 2.25 points, 95% CI, 1.41–3.09, *p* < 0.001). According to the minimum clinically important difference (MCID = 3 points) criterion for ACT scores established by Schatz et al. (2009), the improvement in the observation group (5.05 points) significantly exceeded the MCID threshold, indicating that this improvement has clear clinical significance; whereas the improvement in the control group (2.89 points) did not reach the MCID, and its clinical value remained unclear. The score for the correct use of the inhaler in the observation group was also significantly higher than that in the control group (MD = 4.79 points, 95% CI, 3.08–6.50, *p* < 0.001).

#### 3.2.3 Humanistic value indicators

As shown in Table 5, after 12 months of the intervention, the MARS-A scores in the observation group were significantly higher than those in the control group (MD = 0.44, 95% CI, 0.25–0.63, *p* < 0.001), and a higher proportion of patients demonstrated good adherence (≥4.5 points) (67.2% vs. 43.5%, RR = 1.55, 95% CI, 1.16–2.06, *p* = 0.008). Asthma knowledge scores were also significantly higher in the intervention group than those in the control group (MD = 10.91, 95% CI, 7.05–14.77, *p* < 0.001), and a higher proportion achieved an excellent score (≥80 points) (67.2% vs. 46.8%, RR = 1.43, 95% CI, 1.06–1.92, *p* = 0.022).

## 4 Discussion

### 4.1 Necessity of establishing an asthma T-MTM graded service

Research both domestically and internationally (Lettieri and Cristina, 2009) has amply demonstrated that pharmacist-led MTM services provide significant benefits for patients with chronic airway diseases. A meta-analysis (Lin et al., 2024) showed that MTM services can reduce the risk of acute exacerbations in asthma patients by 30%–50%, improve medication adherence by 20%–40%, and significantly enhance disease knowledge. However, the traditional standardized MTM model is ill-suited to the current shortage of pharmacists in China and suffers from issues such as lengthy processes, excessive workloads, limited service coverage, and waste of resources. Although the existing triangle model is widely used for hypertension and diabetes, there remains a significant lack of systematic, tiered MTM models specifically designed for outpatient asthma care.

In this single-center, prospective randomized controlled trial, we found that the T-MTM model demonstrated a significant advantage over the traditional MTM model in terms of the primary endpoint, i.e., the cost-utility ratio. Additionally, the observation group showed a trend toward better outcomes in secondary endpoints such as ACT scores, medication adherence, and service efficiency.

The marginal contribution of this study does not lie in “pioneering” stratification or MTM but rather in (1) expanding the concept of stratification from “disease severity stratification” to “pharmacy service intensity stratification,” thereby achieving a precise match between patient risk levels and service intensity. (2) For asthma—a specific condition requiring inhalation technique management and medication adherence support—we have established a comprehensive T-MTM practice system (including quantifiable stratification criteria, tiered service content, and multidimensional evaluation indicators). (3) We have filled the evidence gap regarding the systematic application of the triangle stratification model in the field of asthma MTM.

### 4.2 Advantages of risk-based tiered MTM pharmaceutical services

This study used the cost-utility ratio as its primary endpoint. The results showed that the cost-utility ratio in the T-MTM group was significantly lower than that in the conventional MTM group (MD = −7.54, *p* < 0.001). The health economic significance of this difference is reflected on two levels: First is absolute cost savings. The average daily treatment cost in the observation group was 2.33 yuan lower than that in the control group [MD = −2.33 yuan, 95% CI, −3.10 to −1.56]. On an annual basis, each patient receiving T-MTM services could save approximately 213 yuan in treatment costs compared to traditional MTM services (calculated as the daily treatment cost difference of 2.33 yuan/day × 365 days ÷ 4, representing the net savings after deducting the difference in MTM service fees). For patients with chronic asthma requiring lifelong medication, these annual savings have a cumulative effect at both the individual and population levels. Second is the concept of “absolute advantage” in economics. According to pharmacoeconomic evaluation criteria, when a new treatment option is both less expensive and more effective, it is considered an “absolute advantage” option, and its cost-effectiveness can be confirmed without the need for an incremental cost-effectiveness ratio (ICER) threshold assessment. The T-MTM model outperforms the traditional MTM model on both the CUR (lower cost) and ACT score (better efficacy) dimensions, making it a classic example of an absolutely superior option. This means that regardless of the willingness-to-pay threshold adopted by decision-makers (such as the 1–3 times *per capita* GDP recommended by the WHO), the T-MTM model is deemed the more cost-effective choice.

Clinical significance of the ACT score: in a multicenter study of 4,118 asthma patients, Schatz et al. (2009) established the MClD for the ACT score as 3 points using distribution-and anchor-based methods. The study further demonstrated that a three-point reduction in the ACT score was significantly associated with a 76% increase in the risk of subsequent rescue medication use and a 33% increase in the risk of acute exacerbations. In the observation group of this study, the ACT score improved by 5.05 points, exceeding the MCID threshold by 2.05 points; in contrast, the control group improved by 2.89 points, failing to reach the three-point threshold. This implies that patients receiving T-MTM services not only improved their asthma control status from “partially controlled” (18.16 points) to “well-controlled” (23.21 points) but also met the clinically recognized standard for “meaningful improvement”; in contrast, the clinical significance of the improvement achieved through the traditional MTM model remained uncertain.

The MARS-A scores in the observation group crossed the threshold for “good adherence” (≥4.5 points), achieving a behavioral shift from “irregular medication use” to “regular medication use.” Notably, Class C patients receiving the least frequent service (once every 3 months) had better outcomes than patients in the control group receiving monthly services, suggesting that “reducing unnecessary services,” by empowering patients to self-manage, actually enhance their sense of active participation.

The T-MTM service model defines and quantifies the scope of pharmaceutical services for outpatients with asthma, thereby improving service efficiency and standardization. This study clarified the specific service content, frequency, and methods for the different monitoring levels (A, B, and C), thereby eliminating service variability caused by reliance on experience. The T-MTM service model optimized the average service duration per pharmacist from 54 min per patient visit in the traditional MTM model to 30 min per patient visit (*p* < 0.001), representing an approximately 40% increase in service efficiency. More importantly, this study emphasizes the continuous cultivation of patients’ self-management capabilities throughout their disease progression, as evidenced by the positive correlation between self-management scores and clinical improvement (ACT scores) in the observation group. This is consistent with the findings of Chen et al. (2019). In their tiered management of diabetic foot patients based on the triangle model, which showed that for every 1-unit increase in patient self-efficacy, HbA1c decreased by 0.3% (*p* < 0.05).

The results indicate that the new T-MTM service model better aligns with the medical needs of outpatient asthma patients in multidimensional evaluations of “economic-clinical-humanistic management” compared to the traditional MTM service model.

In summary, economic outcome measures suggest that the T-MTM model may have achieved the dual benefits of saving pharmacists time and reducing costs for patients. Clinical outcome measures indicate a trend toward better outcomes in the observation group, with disease control approaching ideal targets. Regarding humanistic value indicators, patients in the observation group demonstrated higher levels of disease awareness and adherence. This may be related to the T-MTM model’s use of tiered services to enhance patients’ sense of active participation; however, the influence of the Hawthorne effect or patient expectation bias cannot be ruled out.

### 4.3 Limitations of the study

First, this study was designed as a single-center trial, which may limit the generalizability of the results; future multicenter RCTs are needed to validate these findings. Additionally, the weighting of each dimension in the triangle stratification criteria lacks support from large-sample evidence; machine learning algorithms could be utilized in future studies to optimize the stratification model. Second, this study employed an open-label design; due to the interactive nature of the T-MTM service, neither participants nor pharmacists were blinded. This may introduce performance bias and detection bias, particularly for patient-reported outcomes such as ACT scores, medication adherence, and satisfaction. Future multicenter randomized controlled trials with blinded outcome assessment are necessary to confirm these findings. Third, there was an inherent difference in service frequency between the observation and control groups. Although this is a deliberate design feature of the T-MTM model (i.e., on-demand service delivery through stratification), it may objectively influence certain subjective outcome measures (e.g., patient satisfaction and knowledge questionnaires). However, from a methodological perspective, statistically “adjusting” for this difference would result in overcorrection, as service frequency is an intrinsic component of the intervention mechanism rather than an exogenous confounding variable. Future studies could adopt an “active control” design (e.g., the control group receiving services of equal frequency but without stratification) to further isolate the independent effects of the stratification strategy.

### 4.4 Conclusion

In summary, the new T-MTM pharmaceutical service model developed in this study demonstrated the potential to enhance pharmacist efficiency, reduce patient costs, and improve disease control and self-management capabilities in a single-center exploratory study. Analysis of the primary outcome measures revealed that the T-MTM model holds an “absolute advantage” over the traditional MTM model—it is more cost-effective (with annual savings of approximately 213 yuan per person) and more efficacious (with ACT improvement exceeding the MCID). Despite methodological limitations, this model integrates the triangle stratification concept with asthma MTM practice, providing a practical model for constructing stratified service workflows and conducting multidimensional efficacy evaluations to optimize outpatient asthma pharmaceutical services. Future multicenter, double-blind randomized controlled trials are needed to further validate its generalizability.

## Data Availability

The raw data supporting the conclusions of this article will be made available by the authors without undue reservation.
